# Influence of cyclodextrin on the UCST- and LCST-behavior of poly(2-methacrylamido-caprolactam)-co-(*N*,*N*-dimethylacrylamide)

**DOI:** 10.3762/bjoc.10.203

**Published:** 2014-08-21

**Authors:** Alexander Burkhart, Helmut Ritter

**Affiliations:** 1 Institute of Organic Chemistry and Macromolecular Chemistry, Heinrich-Heine-University Duesseldorf, Universitaetsstrasse 1, D-40225 Duesseldorf. Germany, Fax: (+49) 211-811-5840

**Keywords:** cyclodextrin, LCST, lysine, 2-methacrylamido-caprolactam, UCST

## Abstract

The monomer 2-methacrylamido-caprolactam (**4**) was synthesized from methacryloyl chloride (**3**) and racemic α-amino-ε-caprolactam (**2**). Copolymerization of **4** with *N*,*N*-dimethylacrylamide (**5**) was carried out by a free-radical mechanism using 2,2’-azobis(2-methylpropionitrile) (AIBN) as an initiator. The new copolymers show a lower critical solution temperature (LCST) in water and an upper critical solution temperature (UCST) in ethanol, 1-propanol, and 1-butanol. The solubility properties of the copolymers can be influenced significantly by the addition of randomly methylated β-cyclodextrin (CD). The complexation of the copolymers with CD, was confirmed by the use of ROESY-NMR-spectroscopy.

## Introduction

Recently, increasing interest has been spent on thermoresponsive polymer solutions, mainly because of their potential application in the field of drug delivery, gene delivery, or tissue engineering [1–3]. Especially polymers having a lower critical solution temperature (LCST) in water, like poly(*N*-isopropylacrylamide) or modified poly(*N*-vinylpyrrolidone) are extensively described in literature [4–5]. Below the critical temperature (*T*_C_) these polymers are soluble and they become insoluble by heating their solutions above the *T*_C_. In contrast, some polymers forming hydrogen bonds, like poly(acrylic acid) or polymers containing zwitter-ionic groups are soluble in water above a critical temperature (UCST) [6–7]. Only a few reports deal with polymers which have both, LCST- and UCST-behavior in water [8]. The *T*_C_ of a thermoresponsive polymer solution can be altered easily by variation of the chemical structure and to a certain degree by variation of the chain length of the used polymer, also. Interestingly, the *T*_C_-values of suitable polymers in water can also be influenced significantly by supramolecular interactions with cyclodextrin [9]. Accordingly, we described in 2003 for the first time, that the LCST of a poly(*N*-adamantylacrylamide-co-*N*’-isopropylacrylamide) is influenced by CD [10]. Furthermore, the interaction of poly(pseudo-betaines) with CD leads to an UCST-behavior in water [11]. A precise control of *T*_C_ via the addition of “effectors”, that induce polymer–polymer interactions has been described [12]. Recently, we published that CD can shift the UCST of copolymers obtained from *N*-vinylimidazole and adamantyl-modified *N*-vinylimidazole [13]. In the present study we evaluate the thermoresponsive behavior of solutions of polymers containing 2-methacrylamido-caprolactam as a comonomer. The used α-amino-ε-caprolactam is obtained from biomass through a combination of biological fermentation and chemical processes [14].

## Results and Discussion

**Scheme 1:**
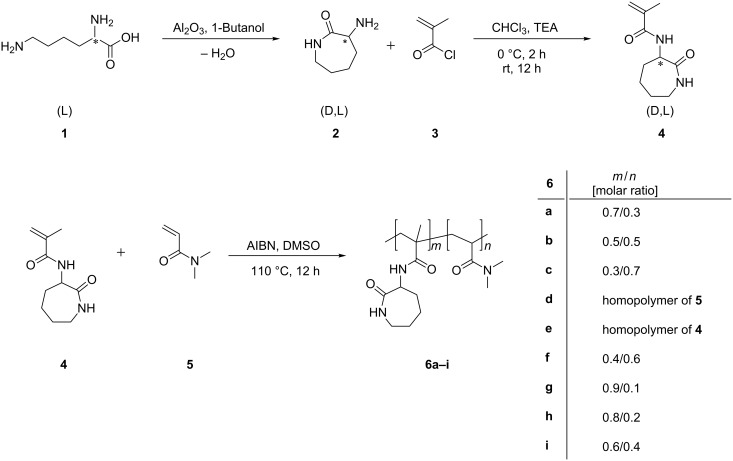
Synthesis of monomer 2-methacrylamido-caprolactam (**4**) and copolymers **6a–i** based on **4** and *N*,*N*-dimethylacrylamide (**5**).

**Figure 1:**
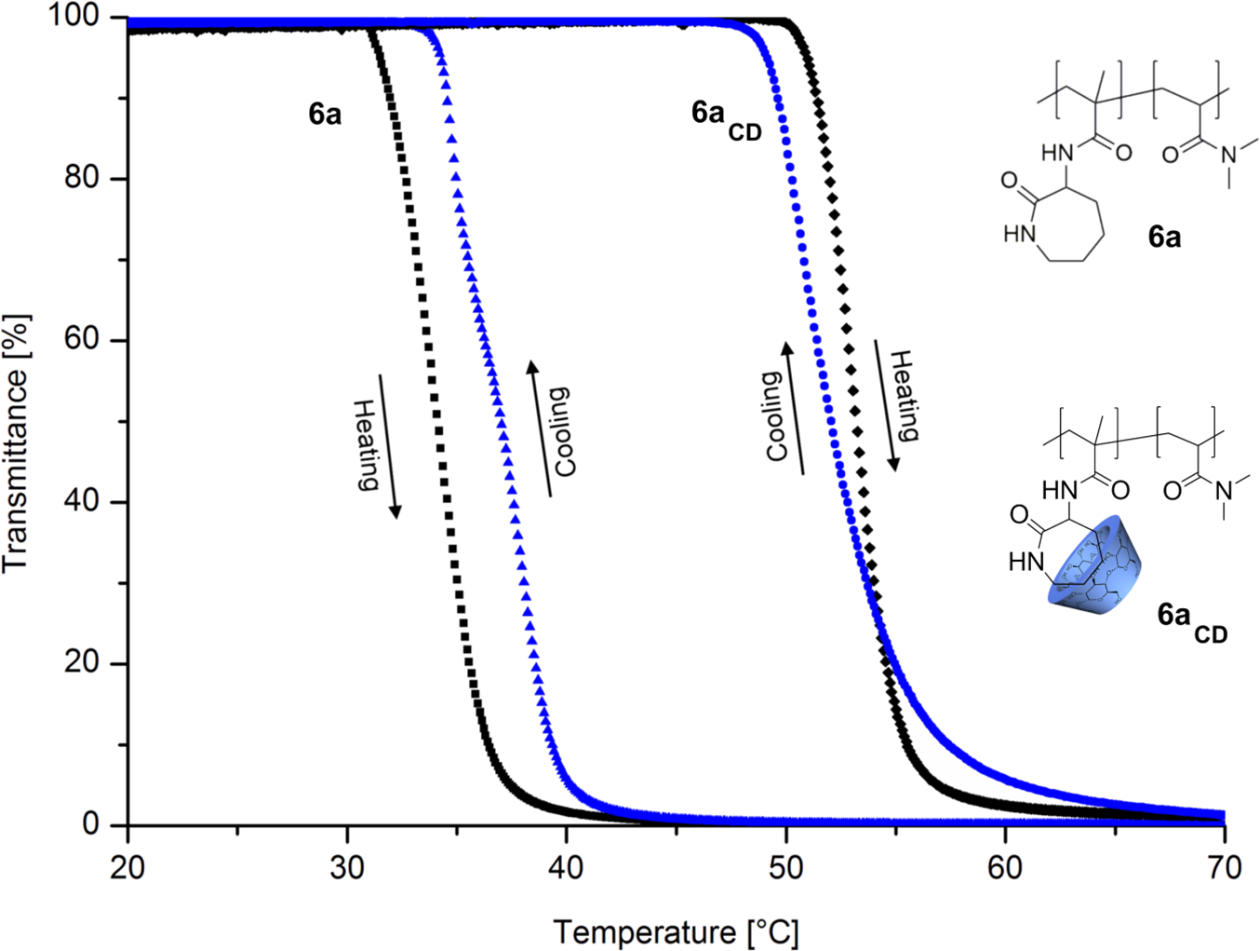
Turbidity curves upon heating and corresponding curves upon cooling of 10 mg ml^−1^ solution of polymer **6a** and **6a_CD_** at a heating/cooling rate of 1 °C min^−1^.

Racemic α-amino-ε-caprolactam (**2**) was obtained according to literature via cyclocondensation of L-lysine (**1**) [15]. Through amidation of the primary amine (**2**) with methacryloyl chloride (**3**) the polymerizable 2-methacrylamido-caprolactam (**4**) was obtained (Scheme 1) [14]. Free radical copolymerization of **4** in various comonomer compositions with *N*,*N*-dimethylacrylamide (**5**) leads to copolymers **6a–i** (Scheme 1). **6a** is soluble in water and methanol and insoluble in ethanol, 1-propanol, and 1-butanol. Copolymers **6b–e** are soluble in water as well as in methanol, ethanol, 1-propanol, and 1-butanol (Figure 1 and Figure 3), respectively. According to ^1^H NMR spectroscopy, the monomer conversions were nearly quantitative. Therefore it is assumed, that the copolymer compositions are exactly identical with the monomer feed ratios.

To investigate the solubility behavior of polymers **6** in water, turbidity measurements were performed. As shown in Figure 1, copolymer **6a** has a typical LCST-behavior in water. The cloud point upon heating is 34 °C and upon cooling 37 °C, respectively. The hysteresis effect is a result of insoluble to soluble transition which depends on the shape and size distribution of dispersed polymer particles [16–17]. The more hydrophilic copolymer **6b** has a higher cloud point at 67 °C whereas copolymer **6c** is completely soluble in water up to >95 °C. Complexation of copolymer **6a** with a 1.5 molar excess of CD, based on monomer **4,** was carried out to yield the complexed copolymer **6a_CD_**. Due to that complexation, the cloud point in the heating curves increases from 34 °C (**6a**) up to 47 °C (**6a_CD_**) with a sharp phase transition. Obviously, the complexation of the attached caprolactam ring with CD leads to a higher hydrophilicity of the copolymer and therefore to a better solubility in water.

**Figure 2:**
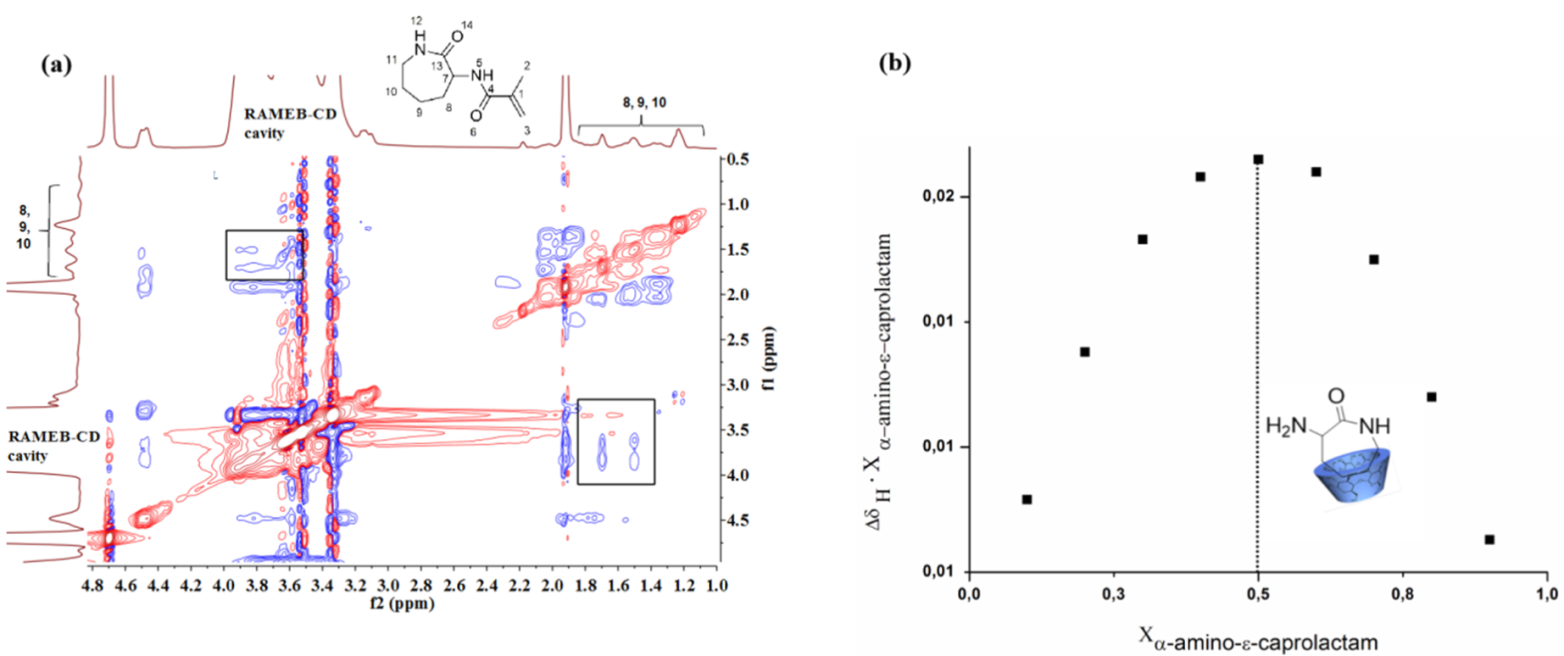
2D NMR ROESY (300 MHz, D_2_O) spectrum of monomer **4_CD_** (a), Job plot of **2** with RAMEB-CD (b).

The postulated complexation of the polymer attached caprolactam moiety in copolymer **6a** with CD was principally demonstrated via ROESY-NMR-spectroscopy in solution using the monomer **4** (Figure 2a). The magnetic interaction of the protons of the cyclodextrin with protons from the lactam ring between 1.1 ppm and 2.0 ppm confirms the complexation. Furthermore, a Job plot has been carried out to identify the stoichiometry of the complex between the caprolactam and the CD. As shown in Figure 2b, a 1:1 complex was found.

To prove that the increase of the *T*_C_ (Figure 1) is a result of complexation with added CD and not a simple solvent effect, α-D(+)-glucose was added to copolymer **6a** in an equimolar amount to the caprolactam groups. As expected, turbidity measurements indicate, that there is no significant increase of the *T*_C_.

**Figure 3:**
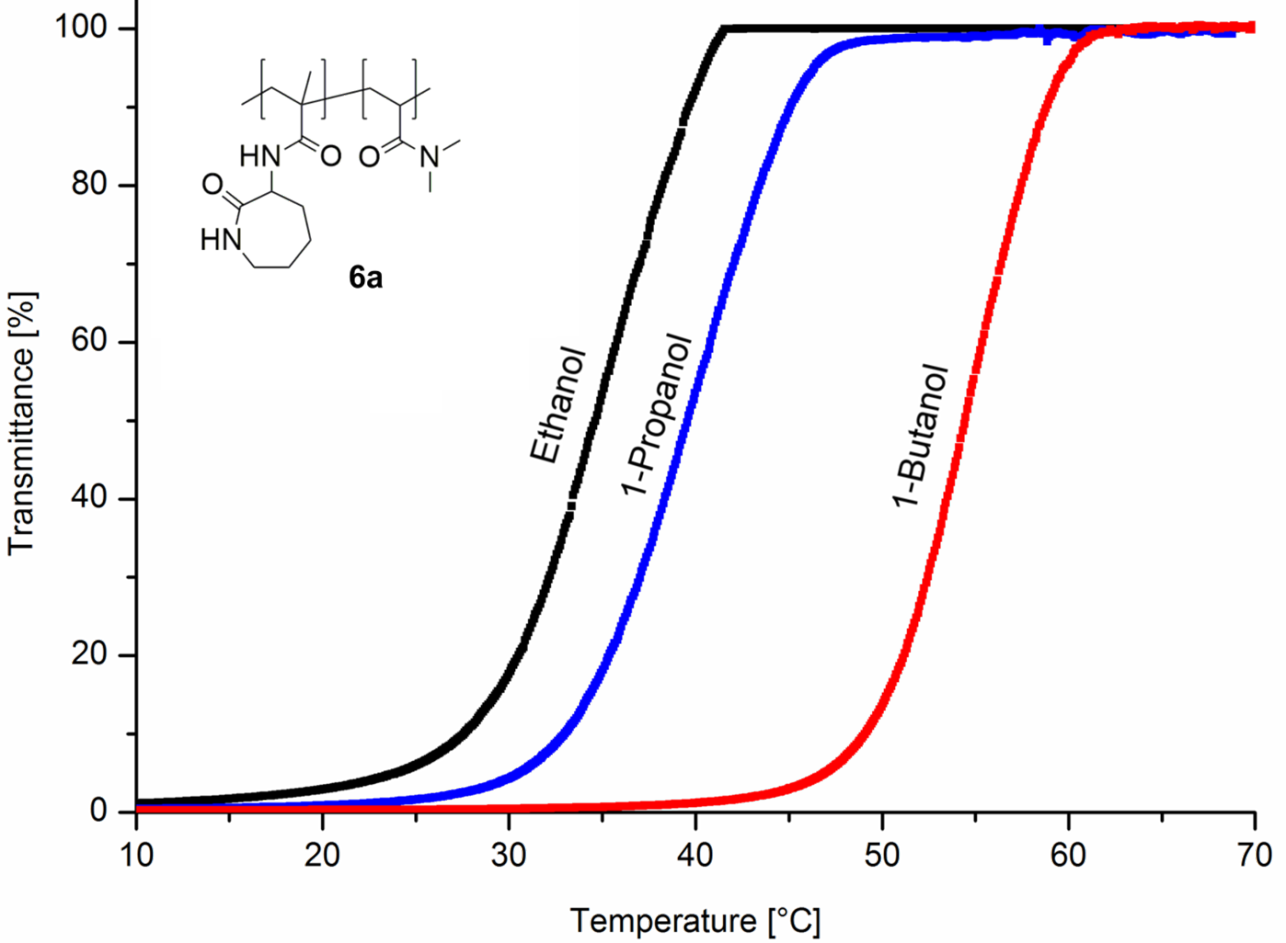
Turbidity curves upon cooling of 10 mg ml^−1^ solution of copolymer **6a** in various *n*-alcohols at a cooling rate of 1 °C min^−1^.

Furthermore, it was found that the copolymer **6a** is completely soluble in methanol. However, in less polar ethanol, 1-propanol, and 1-butanol heating is required to obtain solutions (Figure 3).

**Figure 4:**
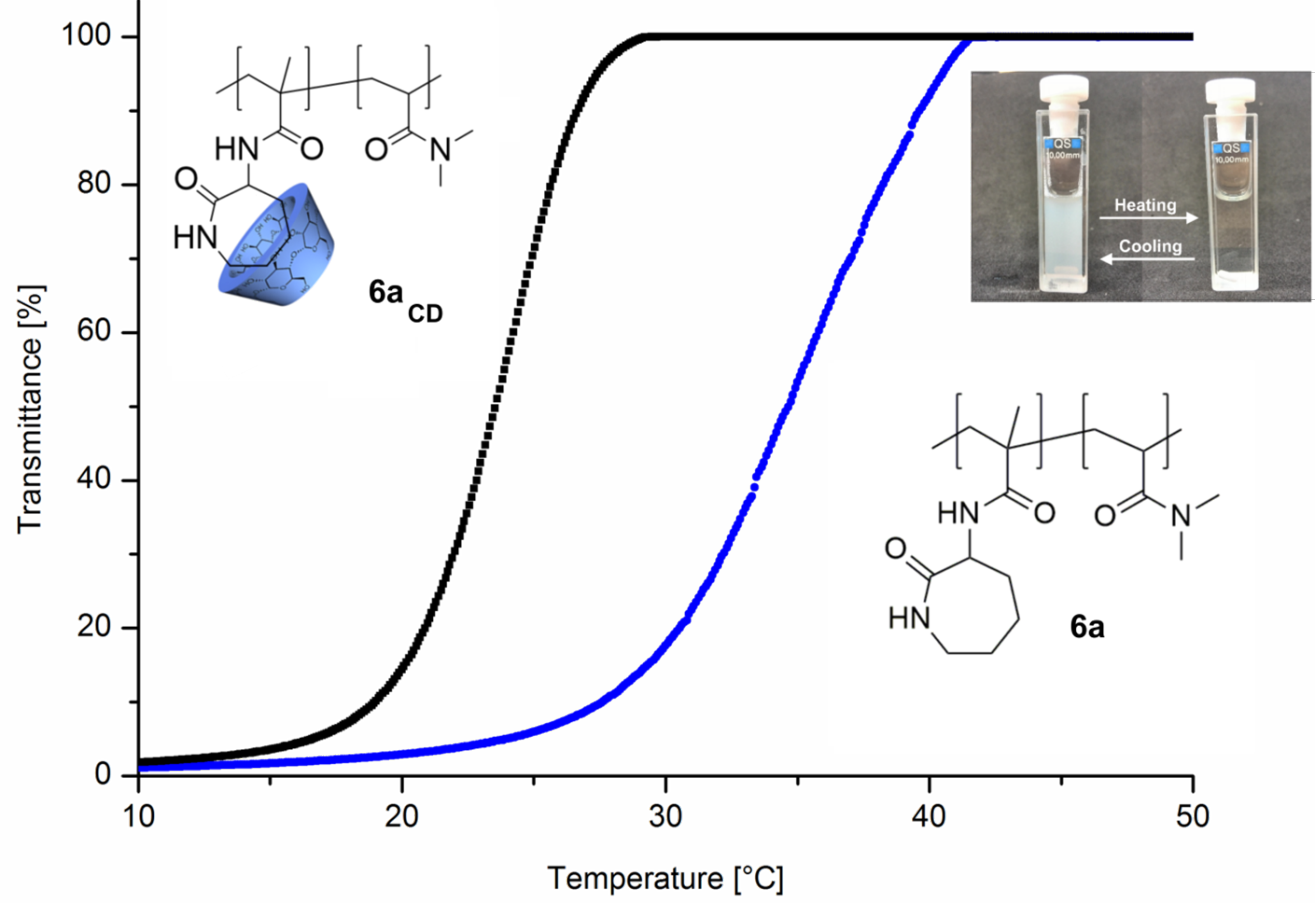
Turbidity curves upon cooling of 10 mg ml^−1^ solution of polymer **6a** and **6a_CD_** at a cooling rate of 1 °C min^−1^ in ethanol.

The observed cloud points of copolymer **6a** in alcohol solution increase from ethanol (35 °C), via 1-propanol (40 °C) up to less polar 1-butanol (54 °C). Since the content of hydrogen-bond forming amido-caprolactam units in copolymers **6b** and **6c** is reduced, the copolymers obviously become completely soluble in the used alcohols in the whole temperature range. The potential influence on the UCST-behavior by complexation with CD of polymer **6a** in ethanol was evaluated by turbidity measurements (Figure 4).

**Figure 5:**
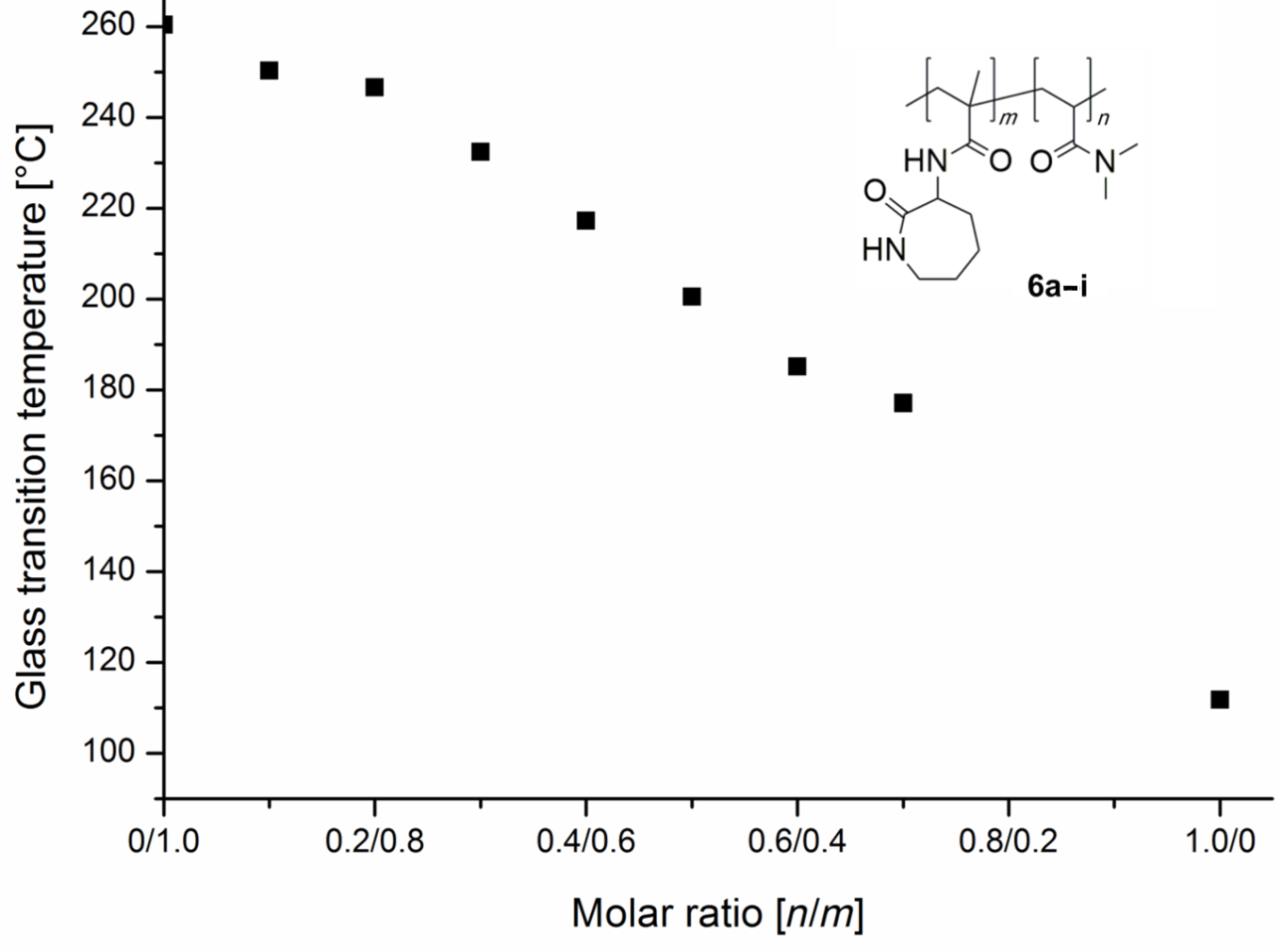
Glass-transition temperature as a function of the amount of *N*,*N*-dimethylacrylamide (**5**) in the copolymers **6a–i**.

Actually, the cloud point of copolymer **6a** shifts from 34 °C down to 24 °C in ethanol, because of **6a_CD_** formation. Due to that complexation of the polymer attached caprolactam-rings by CD the number of intermolecular hydrogen bonds is reduced which leads to a better solubility in the used alcohols, as mentioned above. The amount of CD was varied from 50 mol % up to 150 mol % based on the amount of caprolactam units of copolymer **6a**. Surprisingly this variation of CD amount did not significantly influence the cloud point (24 °C) of copolymer **6a_CD_**. Obviously, the dense packing of the caprolactam moieties in the copolymer allows the approach of the bulky CD rings only partially up to a certain limit to form those complexes. Molecular weights of copolymer **6a** could not yet be detected by the use of gel-permeation chromatography (GPC) even under various conditions, probably due to strong interactions of the amide groups of the copolymer. Besides that, the measured hydrodynamic coil size of about 100 nm via dynamic light scattering (DLS) in different solutions indicates the formation of agglomerates. We also spent some interest in the thermal behavior of copolymers **6** in the condensed state. The observed glass-transition temperatures (*T*_g_) of copolymers **6a–i** in condensed phase, are summarized in Figure 5.

Generally with the increasing amount of flexible *N*,*N*-dimethylacrylamide (**5**) in the copolymer chains, the glass transition temperatures are decreasing, as expected. Due to the stronger intermolecular hydrogen-bond interactions in the relatively stiff homopolymer **6e** the glass-transition temperature is higher than in the copolymers with various amounts of *N*,*N*-dimethylacrylamide (**5**).

## Conclusion

It can be concluded from the above described results, that the novel copolymers from lysine based 2-methacrylamido-caprolactam (**4**) and *N*,*N*-dimethylacrylamide (**5**) show LCST-behavior in water and UCST-behavior in certain alcohols also, which can be controlled by the monomer ratios. Surprisingly, the complexation of the caprolactam-bearing units in the copolymer with CD leads to an increase of LCST values in water and vice versa, to a decrease of UCST in ethanol. According to that, these novel copolymers are of potential interest for the development of new types of smart materials.

## Experimental

### 

#### Materials and methods

All reactants were commercially available and used without further purification. All solvents used were of analytical purity or freshly distilled. L(+)-lysine monohydrochloride (99+%) was purchased from Acros Organics and *N*,*N*-dimethylacrylamide (99%) was obtained from Sigma-Aldrich. Methacryloyl chloride ( ≥99%) and triethylamine pure (99%) were purchased from AppliChem. RAMEB-CD was obtained from Wacker Chemie GmbH, Burghausen, Germany and was used after being dried with a vacuum oil pump over P_4_O_10_. 2,2’-Azobis(2-methylpropionitrile) (99%) was obtained from Sigma-Aldrich. α-D(+)-glucose (99+%) was obtained from Acros Organics.

^1^H NMR and ^13^C NMR spectra were recorded on a Bruker Avance DRX 300 by using dimethyl sulfoxide-*d*_6_ or chloroform-*d* as solvents. The chemical shifts (δ) are given in parts per million (ppm) using the solvent peak as an internal standard. 2D NMR-spectroscopy was performed using a Bruker Avance III DRX 300 at 300 MHz in D_2_O as a solvent. The samples were measured at room temperature. FTIR spectra were recorded on a Nicolet 6700 FTIR spectrometer equipped with an ATR unit. Differential scanning calorimetry (DSC) was performed using a Mettler Toledo DSC 822 instrument equipped with a sample robot TSO801RO. For calibration, standard tin, indium, and zinc samples were used. For *T*_g_ determination, heating and cooling curves were recorded between −50 and 330 °C at a heating rate of 15 K min^−1^. The *T*_g_ value was calculated from the arithmetic average of the inflection points of the second, third, and fourth heating curve. Turbidity measurements were accomplished using a TP1 turbidity photometer in a temperature range of 5 to 70 °C. During continuous stirring, the transparency of the sample was determined by a voltage-controlled semiconductor or laser and a silicon photodiode at a wavelength of 590 nm and a heating or cooling rate of 1 °C min^−1^. The critical temperature was determined at 50% of relative transmittance. Electrospray ionization mass spectrometry (ESIMS) was conducted on a Bruker maXis 4G mass spectrometer. Melting points were obtained using a Büchi Melting Point B-545 apparatus at a heating rate of 1 °C/min.

#### Synthesis of α-amino-ε-caprolactam (**2**)

A mixture of 40 g (219 mmol) of L-lysine monohydrochloride (**1**), 8.76 g (219 mmol) sodium hydroxide, 120 g (1.178 mol) aluminium oxide and 450 ml of *n*-butanol is heated to reflux for 48 h in a reaction vessel equipped with a water trap. Subsequently, the mixture is filtrated and the obtained pale yellow solution is concentrated under reduced pressure. After precipitation in diethyl ether, filtration and drying in vacuum 15.6 g (121 mmol, 55.6%) of colorless/pale yellow crystals are received. Mp 63–67 °C; FTIR (diamond), ν (cm^−1^): 3355, 3282 (br. NH), 2928 (s, CH), 2909 (s, CH), 2848 (m, CH), 1648 (s, amide), 1567, 1474, 1431, 1316; ^1^H NMR (300 MHz, CDCl_3_) δ 6.52 (s, 1H, NH), 3.51 (dd, *J* = 10.8, 2.0 Hz, 1H, CH), 3.19 (m, 2H, RHN-CH_2_-R), 2.1–1.3 (m, 6H, (CH_2_)_3_); ^13^C NMR (75 MHz, CDCl_3_) δ 179.65 (1C, *C*(O)), 54.06 (1C, *C*H), 42.04 (1C, RHN-*C*H_2_-R), 33.97 (1C, RHC-*C*H_2_-R), 29.20 (1C, *C*H_2_), 28.57 (1C, *C*H_2_); ESIMS (H_2_O) *m*/*z*: 129.1 [M + H]^+^.

#### Synthesis of 2-methacrylamido-caprolactam (**4**)

In a 100 mL round bottom flask 5 g (39.01 mmol) of α-amino-ε-caprolactam (**2**) is dissolved in 50 mL of dry chloroform. 7.89 g (78.02 mmol) of triethylamine is added and the solution is cooled in an ice bath to 0 °C. To this solution 4.89 g (46.8 mmol) of methacryloyl chloride (**3**), dissolved in 20 ml anhydrous chloroform, is added within 10 min under nitrogen atmosphere. The mixture is stirred for further 2 h at 0 °C, before warming up to room temperature and stirring for additional 12 h. Then the reaction mixture is washed with brine and water. The organic phase is dried over magnesium sulfate, filtered and the solvent is evaporated under reduced pressure. A colorless solid is obtained. Mp = 125 °C; Yield: 4.7 g (23.9 mmol, 61%); FTIR (diamond), ν (cm^−1^): 3252 (br. NH), 2923 (s, CH), 2857 (m, CH), 1650 (s, amide), 1513, 1476, 1332, 1029; ^1^H NMR (300 MHz, CDCl_3_) δ 6.29 (s, 1H, NH), 5.79 (p, *J* = 1 Hz, 1H, CH_2_), 5.35 (p, *J* = 1.5 Hz, 1H, CH_2_), 3.40–3.15 (m, 3H, RHN**-**CH_2_-R, CH), 2.35–1.2 (m, 9H, (CH_2_)_3_, CH_3_); ^13^C NMR (75 MHz, CDCl_3_) δ 174.21 (1C, *C*(O)), 166.04 (1C, NH**-***C*(O)-C), 139.57 (1C, R**-***C*=CH_2_), 119.48 (1C, H_2_*C*=C**-**R), 51.54 (1C, *C*H), 40.63 (1C, RHN**-***C*H_2_-R), 30.78 (1C, RHC**-***C*H_2_-R), 28.83 (1C, *C*H_2_), 27.66 (1C, *C*H_2_), 18.38 (1C, *C*H_3_); ESIMS (ethanol) *m*/*z*: 197.1 [M + H]^+^, 219.1 [M + Na]^+^.

#### General procedure for the preparation of copolymers **6a–i**

The monomer 2-methacrylamido-caprolactam (**4**), was mixed in different molar ratios (according to Table 1) with *N*,*N*-dimethylacrylamide (**5**) in dimethyl sulfoxide (total monomer concentration 1.8 mol L^−1^). 1 mol % of 2,2’-azobis(2-methylpropionitrile) with respect to monomers **4** and **5** were added to the solution under nitrogen. Under stirring, the solution was heated to 110 °C. After 12 h, the polymerization was stopped by cooling the solution to room temperature. The copolymers were precipitated in ethyl acetate, filtered off and dried in vacuo. The obtained products are colorless solids. Analytical data refer to monomer ratio of 1:1. FTIR (diamond), ν (cm^−1^): 3392 (br. NH), 2929 (CH), 1632 (s, amide), 1476, 1435, 1333, 1136; ^1^H NMR (300 MHz, DMSO-*d*_6_) δ 7.96 (s, 1H, NH), 7.21 (s, 1H, NH), 4.23 (s, 1H, CH), 3.3-2.6 (m, 11H, RHN-C*H*_2_-R, N-C*H*_3_, C*H*_2_ backbone, C*H*, backbone), 2.1–0.6 (m, 11H, C*H*_2_, C-C*H*_3_, C*H*_2_).

**Table 1:** Monomer amount of **4** and **5** for the copolymerization of copolymers **6a–i**.

Polymer **6**	2-methacrylamido- caprolactam (**4**)		*N*,*N*-dimethylacryl- amide (**5**)	
	
	[mmol]	[g]	[mmol]	[g]

**a**	2.5	0.5	1	0.1
**b**	2.5	0.5	2.5	0.25
**c**	2.5	0.5	5.8	0.57
**d**	0	0	2.5	0.25
**e**	2.5	0.5	0	0
**f**	2.5	0.5	0.3	0.03
**g**	2.5	0.5	0.6	0.06
**h**	2.5	0.5	1.7	0.17
**i**	2.5	0.5	3.8	0.38

#### General Procedure for the preparation of the standard solutions for the Job Plot analysis

0.16 g of α-amino-ε-caprolactam is dissolved in 10 ml water to achieve a 0.125 M standard solution (**A**). A second standard solution (**B**) is received by dissolving 1,64 g RAMEB cyclodextrin in 10 ml water, 0.125 M. Both solutions were stirred for 24 h before mixing them together in different amounts (Table 2). These solutions are stirred for additional 24 h before recording an ^1^H NMR.

**Table 2:** Different amounts of standard solution (**A**) and (**B**).

0.125 M α-amino-ε-caprolactam solution (**A**)	0.125 M RAMEB-cyclodextrin solution (**B**)

0	1
0.1	0.9
0.2	0.8
0.3	0.7
0.4	0.6
0.5	0.5
0.6	0.4
0.7	0.3
0.8	0.2
0.9	0.1

## Supporting Information

File 1 Characterization data of intermediates and copolymers including ^1^H NMR and ^13^C NMR spectra, turbidity- and DLS-measurements.
