# Supplementary material for: Influence of cyclodextrin on the UCST- and LCST-behavior of poly(2-methacrylamido-caprolactam)-co-(N,N-dimethylacrylamide)
Source: Beilstein J Org Chem. 2014 Aug 21;10:1951–8. doi: 10.3762/bjoc.10.203 (PMC4168891; doi:10.3762/bjoc.10.203)
Supplement: File 1 — Characterization data of intermediates and copolymers including 1H NMR and 13C NMR spectra, turbidity- and DLS-measurements. [file Beilstein_J_Org_Chem-10-1951-s001.pdf]

Supporting Information

for

Influence of cyclodextrin on the UCST- and LCST-behavior of poly(2-methacrylamido-caprolactam)-co-(*N*,*N*-dimethylacrylamide)

Alexander Burkhart, Helmut Ritter*

Address: Institute of Organic Chemistry and Macromolecular Chemistry, Heinrich-Heine-University Duesseldorf, Universitaetsstrasse 1, D-40225 Duesseldorf. Germany, Fax: (+49) 211-811-5840

Email: Helmut Ritter* – h.ritter@uni-duesseldorf.de

* Corresponding author

Characterization data of intermediates and copolymers including ^{1}H NMR and ^{13}C NMR spectra, turbidity- and DLS-measurements.

Content:

- Turbidity measurement of copolymer **6b**.
- Determination of the hydrodynamic diameter of copolymer **6a**.
- ^{1}H NMR spectrum of 2-methacrylamido-caprolactam (**4**).
- ^{13}C NMR spectrum of 2-methacrylamido-caprolactam (**4**).
- IR spectrum of 2-methacrylamido-caprolactam (**4**) in solid state.
- ^{1}H NMR spectrum of poly(2-methacrylamido-caprolactam)-co-(*N*,*N*-dimethylacrylamide) **6b**.
- IR spectrum of poly(2-methacrylamido-caprolactam)-co-(*N*,*N*-dimethylacrylamide) **6b** in the solid state.
- ^{1}H NMR spectra of complex between α-amino-ε-caprolactam **(2)** and RAMEB-CD for the Job plot analysis.

Turbidity measurement of copolymer **6b**

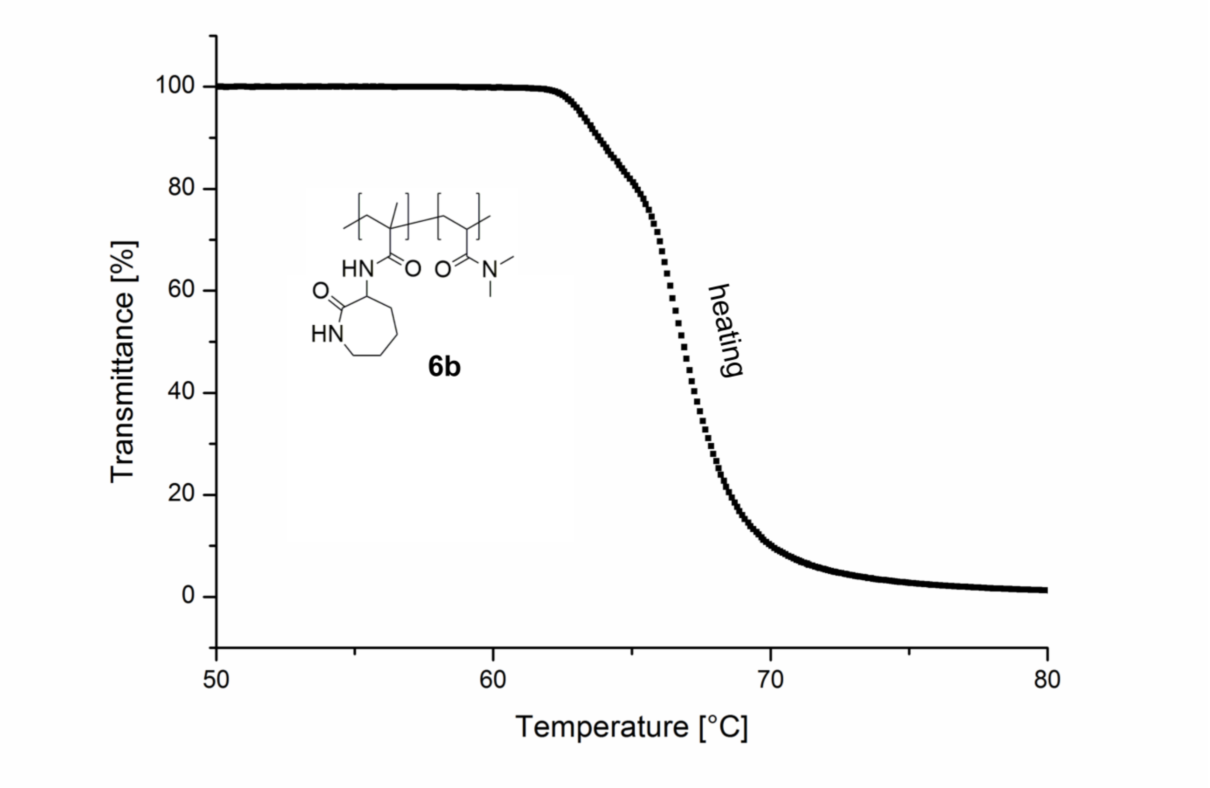

Figure S1: LCST measurements of copolymer **6b** in water upon heating of 10 mg/ml at a heating rate of 1 °C min^{-1}.

Determination of the hydrodynamic diameter of copolymer **6a**

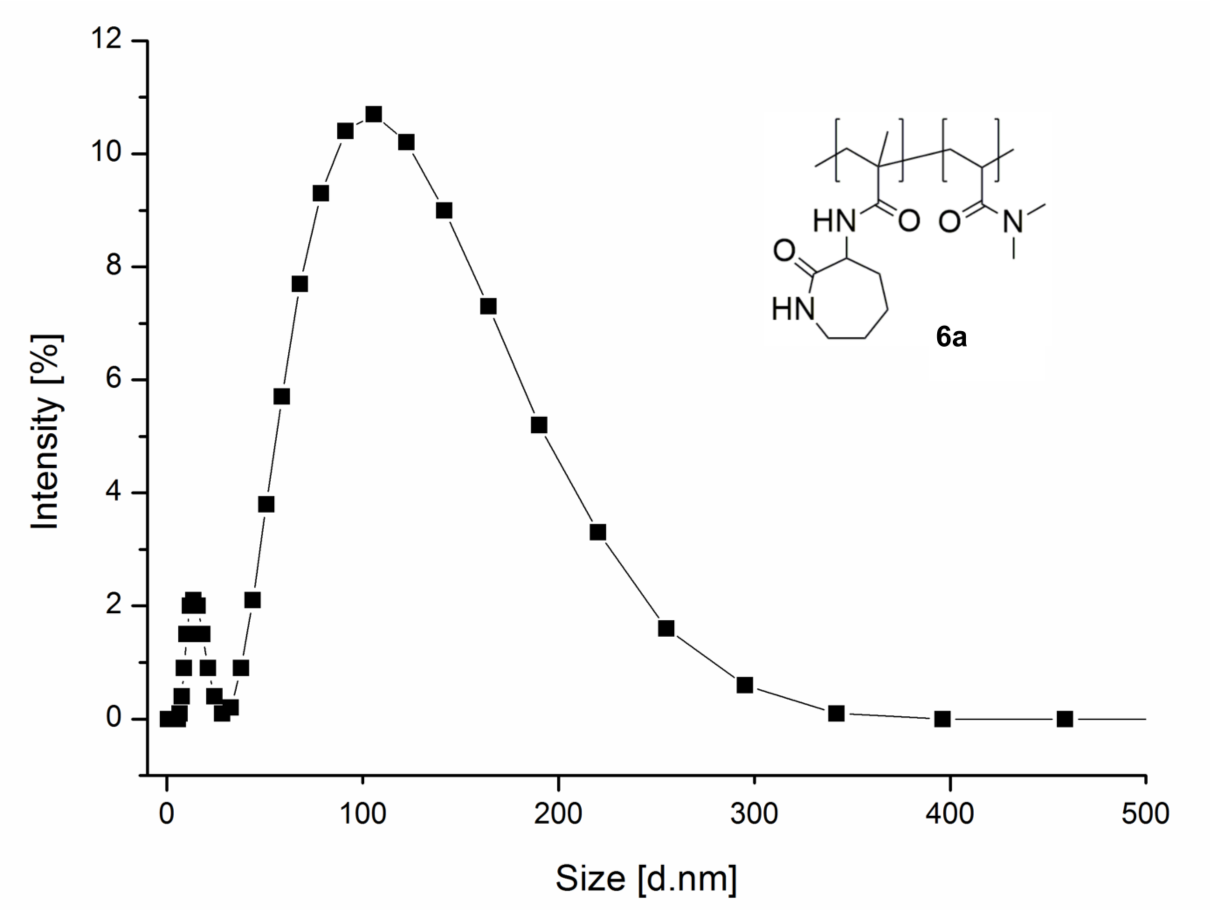

Figure S2: Hydrodynamic diameters of **6a** in ethanol (2 mg ml^{-1}) at 20 °C.

^{1}H NMR spectrum of 2-methacrylamido-caprolactam (**4**)

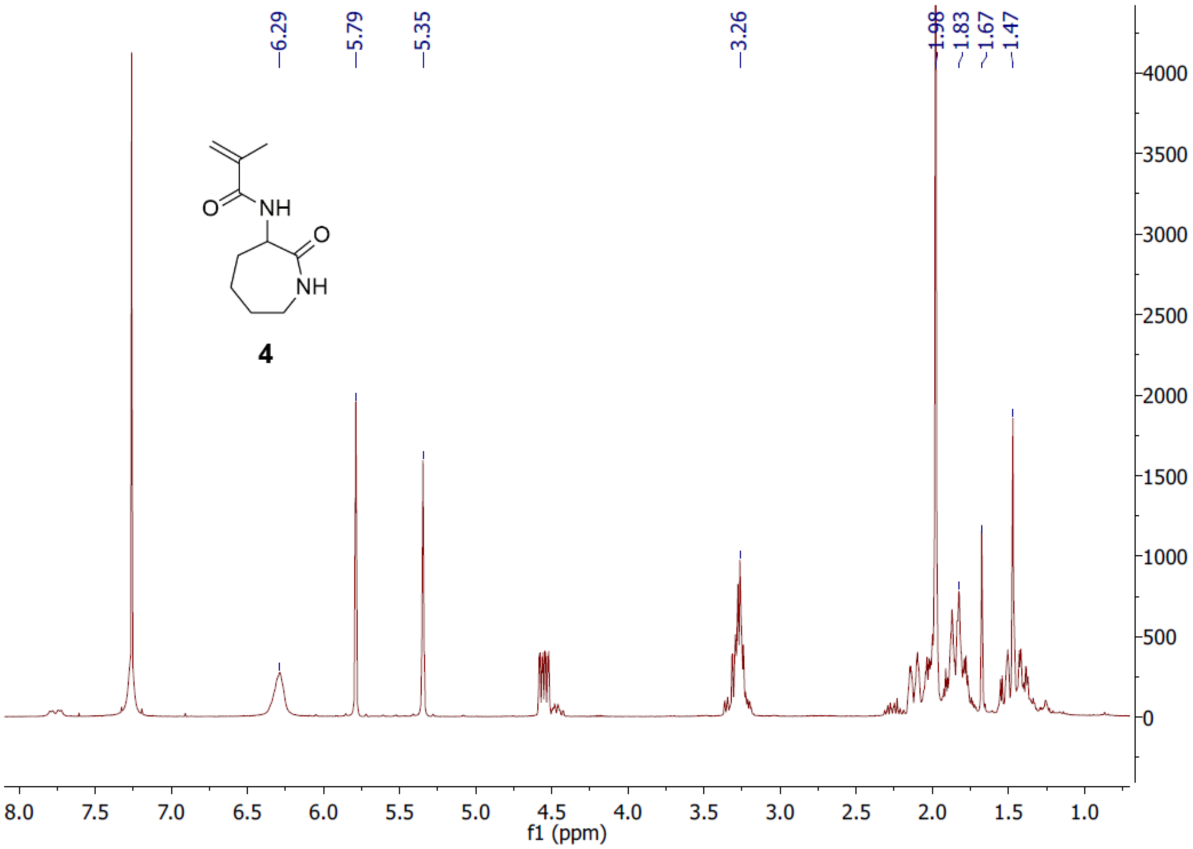

Figure S3: ^{1}H NMR spectrum of 2-methacrylamido-caprolactam (**4**) in $CDCl_3$.

^{13}C NMR spectrum of 2-methacrylamido-caprolactam (**4**)

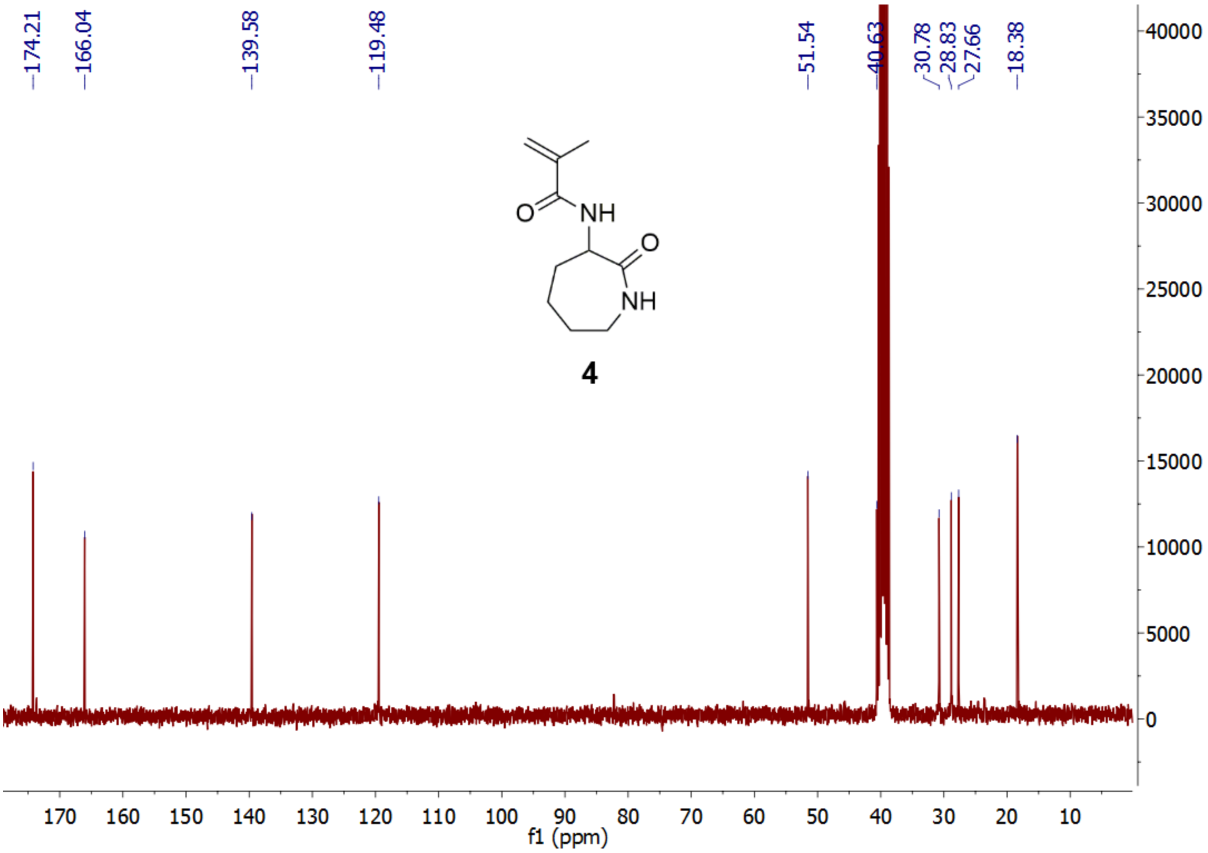

Figure S4: ^{13}C NMR spectrum of 2-methacrylamido-caprolactam (**4**) in dimethylsulfoxide-d_6.

IR spectrum of 2-methacrylamido-caprolactam (**4**) in the solid state

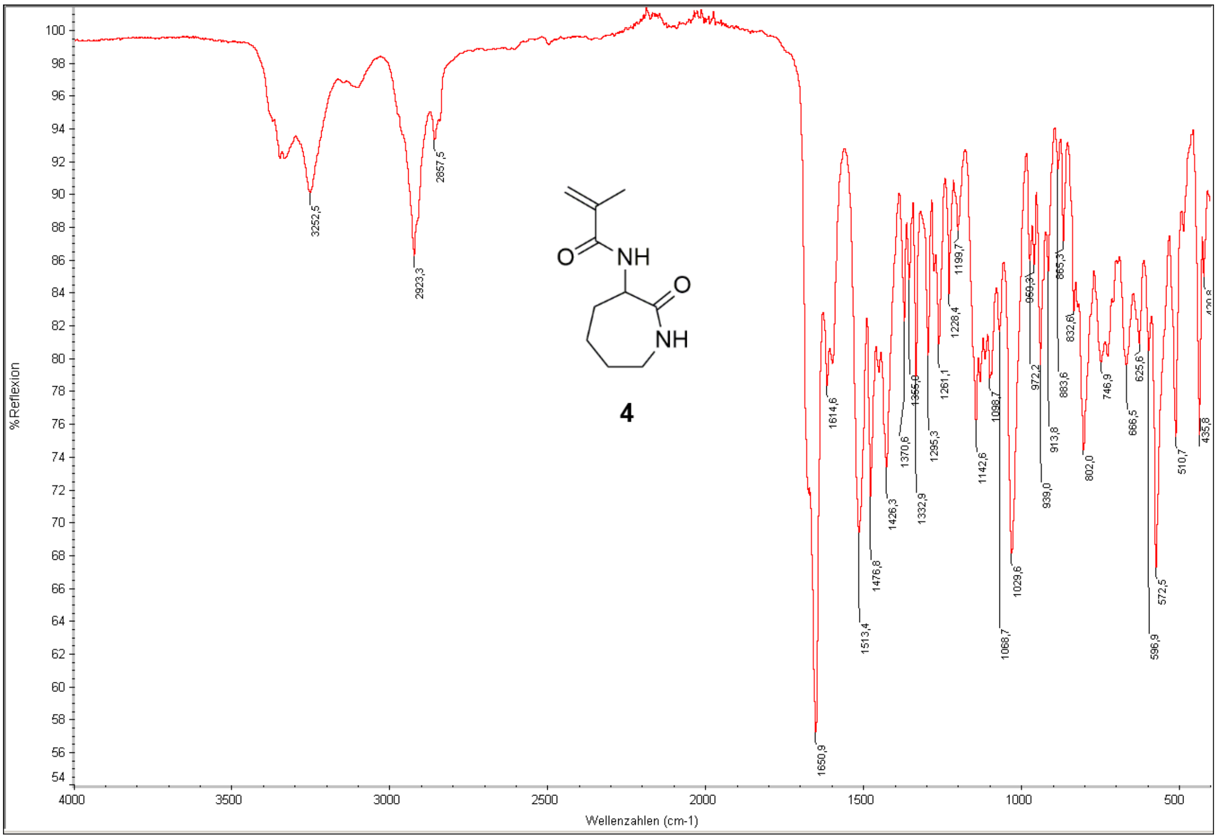

Figure S5: IR spectrum of 2-methacrylamido-caprolactam (**4**) in the solid state.

^{1}H NMR spectrum of poly(2-methacrylamido-caprolactam)-co-(*N*,*N*-dimethylacrylamide) **6b**

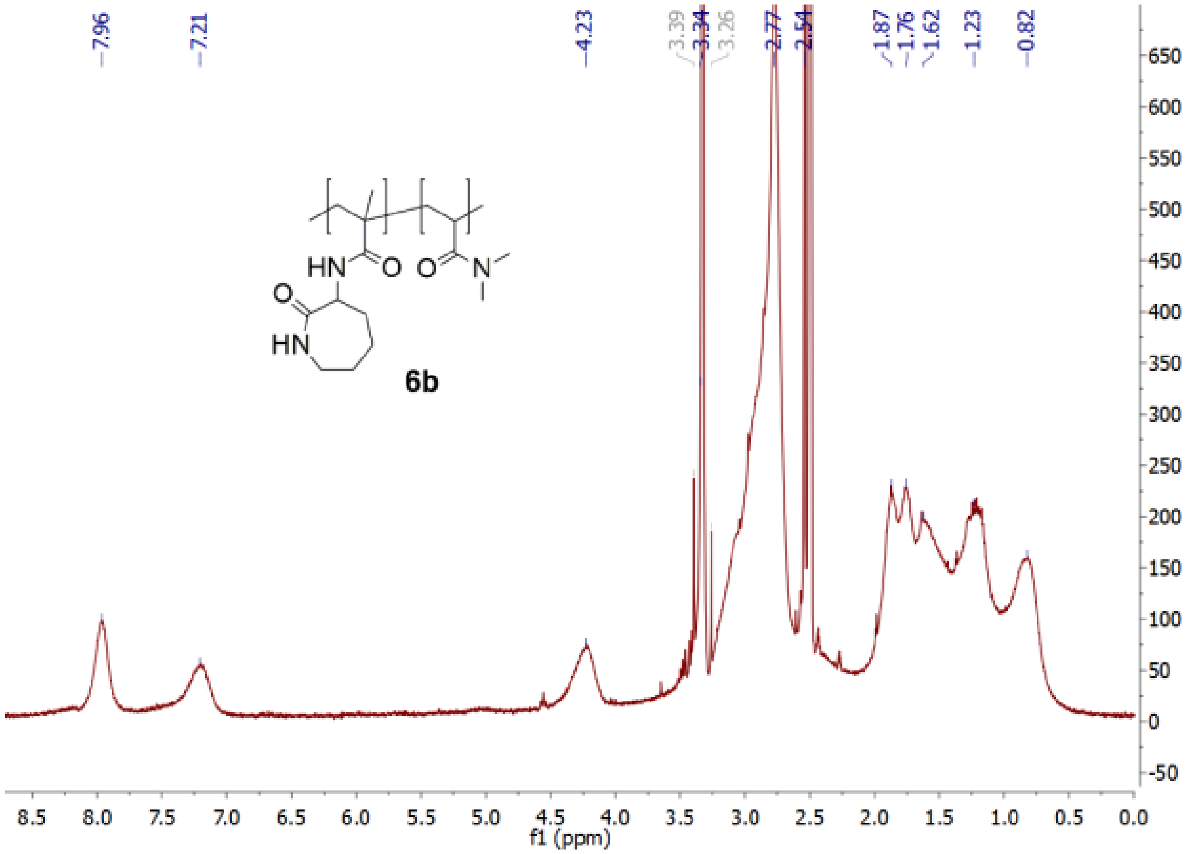

Figure S6: ^{1}H NMR spectrum of copolymer **6b** in dimethylsulfoxide-d_6.

IR spectrum of poly(2-methacrylamido-caprolactam)-co-(*N*,*N*-dimethylacrylamide) **6b**

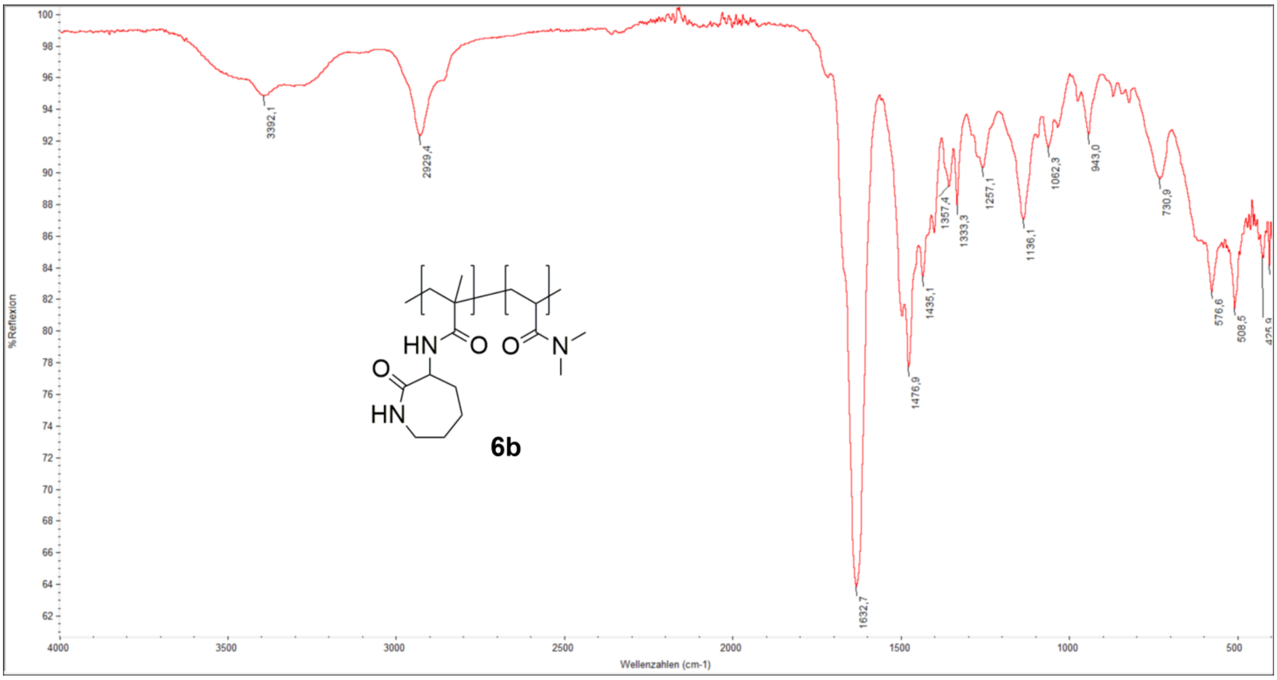

Figure S7: IR spectrum of copolymer **6b** in the solid state.

^{1}H NMR spectra of Job plot analysis of RAMEB cyclodextrin with α-amino-ε-caprolactam (**2**)

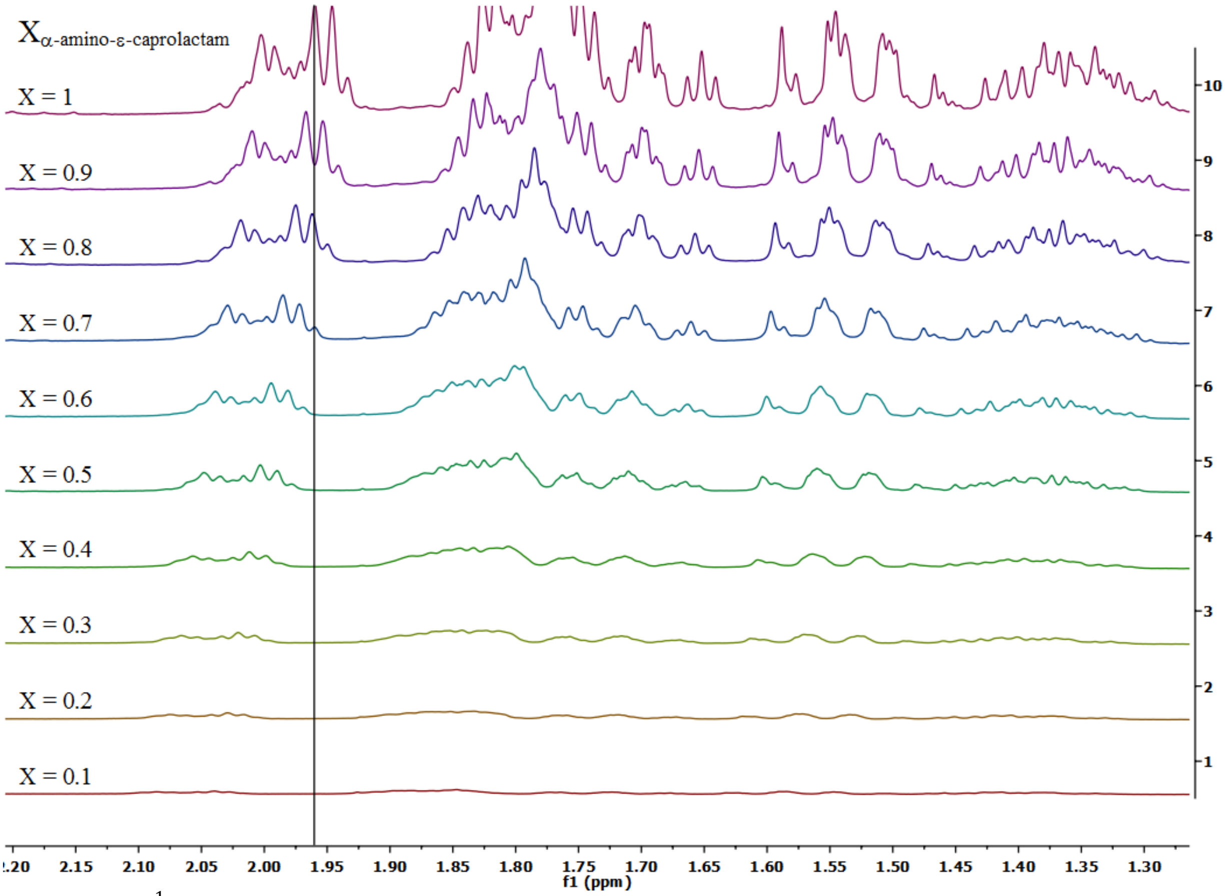

Figure S8: ^{1}H NMR spectra of complex between RAMEB cyclodextrin and α-amino-ε-caprolactam (**2**) in D_2O.
